# Behavioral metabolomics: how behavioral data can guide metabolomics research on neuropsychiatric disorders

**DOI:** 10.1007/s11306-023-02034-6

**Published:** 2023-08-02

**Authors:** Ross van de Wetering, Jan A. Vorster, Sophie Geyrhofer, Joanne E. Harvey, Robert A. Keyzers, Susan Schenk

**Affiliations:** 1 School of Psychology, Victoria University of Wellington, Wellington, New Zealand; 2 School of Chemical and Physical Sciences, Victoria University of Wellington, Wellington, New Zealand

**Keywords:** Metabolomics, Behavior, Addiction, MDMA, Sensitization, LCMS

## Abstract

**Introduction:**

Metabolomics produces vast quantities of data but determining which metabolites are the most relevant to the disease or disorder of interest can be challenging.

**Objectives:**

This study sought to demonstrate how behavioral models of psychiatric disorders can be combined with metabolomics research to overcome this limitation.

**Methods:**

We designed a preclinical, untargeted metabolomics procedure, that focuses on the determination of central metabolites relevant to substance use disorders that are (a) associated with changes in behavior produced by acute drug exposure and (b) impacted by repeated drug exposure. Untargeted metabolomics analysis was carried out on liquid chromatography-mass spectrometry data obtained from 336 microdialysis samples. Samples were collected from the medial striatum of male Sprague-Dawley (*N* = 21) rats whilst behavioral data were simultaneously collected as part of a (±)-3,4-methylenedioxymethamphetamine (MDMA)-induced behavioral sensitization experiment. Analysis was conducted by orthogonal partial least squares, where the *Y* variable was the behavioral data, and the *X* variables were the relative concentrations of the 737 detected features.

**Results:**

MDMA and its derivatives, serotonin, and several dopamine/norepinephrine metabolites were the greatest predictors of acute MDMA-produced behavior. Subsequent univariate analyses showed that repeated MDMA exposure produced significant changes in MDMA metabolism, which may contribute to the increased abuse liability of the drug as a function of repeated exposure.

**Conclusion:**

These findings highlight how the inclusion of behavioral data can guide metabolomics data analysis and increase the relevance of the results to the phenotype of interest.

## Introduction

There is an urgent need for new prevention and treatment strategies that can reduce the significant medical, financial, and emotional burden caused by substance use disorders (SUDs). It is now well accepted that SUDs are disorders of the brain, and some of the brain mechanisms that are altered by repeated exposure to drugs that are misused have been identified. Treatments that have targeted these mechanisms have not, however, been effective for many SUDs (Forray & Sofuoglu, 2014; Nestler, 2022; Volkow et al., 2016). Novel and more efficient means to identify relevant brain mechanisms that are altered by repeated drug exposure might lead to more effective treatment avenues.

Metabolomics is the large-scale analysis of small molecules (< 1500 Da) within cells, biofluids, tissues, or organisms (Fiehn, 2002; Mamas et al., 2011; Patti et al., 2012; Shulaev, 2006; Zhou et al., 2012). There are two main approaches within metabolomic research: targeted and untargeted. Targeted metabolomics quantitatively measures a limited number of known metabolites and is carried out using a list of target analytes and prepared external standards. Since the metabolites of interest must be known *a priori*, targeted metabolomics is partially hypothesis-driven, which prevents the discovery of novel or unexpected metabolites that might be relevant. Untargeted metabolomics, in contrast, aims to semi-quantitively measure all metabolites detectable within the sample. Thus, untargeted metabolomics is hypothesis-generating, and can be used as an efficient, high-throughput means for the potential discovery of novel diagnostic, prognostic, or therapeutic biomarkers of disease.

There have been significant advances in metabolomics research recently and both targeted and untargeted approaches have been used in a variety of applications including pharmacology, toxicology, food science and nutrition, and bioengineering (Giera et al., 2022). However, the application of metabolomics for the study of psychiatric disorders, particularly SUD, is still in its infancy. Several studies have been successful in identifying metabolomic changes associated with SUD in human blood/urine samples, or associated with drug exposure in laboratory animals, but the relevance of many of these metabolomic changes is limited (for reviews see Ghanbari and Sumner, 2018; Mussap et al., 2020; Sethi & Brietzke, 2016; Wang et al., 2016; Zaitsu et al., 2016). For example, in human studies, the analysis of peripheral metabolites from blood/urine samples may not provide the best insight into the underlying neurobiological mechanisms of psychiatric disorders such as SUDs. Additionally, studies in human subjects are limited because of the ethical constraints of repeatedly administering drugs with misuse potential and the inability to adequately control for critical environmental and genetic variables. These limitations are not relevant to preclinical studies. Nonetheless, only a small number of preclinical studies have examined the effect of acute versus repeated drug exposure on the metabolome, and little to no information about drug-produced changes in metabolite levels as a function of time have been obtained. Moreover, valuable behavioral data is rarely collected or utilized, which we suggest can be used to guide metabolomics data analysis in order to increase the relevance of the results to the disorder phenotype of interest and improve translation.

To this end, we have designed a preclinical, untargeted metabolomics procedure that focuses on the determination of central metabolites that are (a) associated with changes in behavior produced by acute drug exposure and (b) impacted by repeated drug exposure. A key component of this procedure is the use of microdialysis as the sampling method (Chefer et al., 2009; van Mever et al., 2021; Westerink & Cremers, 2007). Brain microdialysis involves the perfusion of artificial cerebrospinal fluid through a small probe equipped with a semi-permeable membrane that has been implanted into the brain region of interest. Neurotransmitters, metabolites, and other small molecules present in the extracellular space diffuse across this membrane and into the perfusate, which is subsequently analyzed for its constituents, typically by high performance liquid chromatography (HPLC) coupled with various detectors. As a sampling procedure for metabolomics, microdialysis offers several important advantages. First and foremost, microdialysis permits neurochemical samples to be collected from awake, freely moving subjects, which allows for the concurrent collection of valuable behavioral data. Second, microdialysis provides important temporal information since samples can be collected every ~ 5–30 min. Lastly, microdialysis only samples the extracellular fluid from a relatively discrete brain region, and therefore, offers results that are much more relevant to neurotransmission and behavior compared to whole tissue analysis.

As a proof of concept, we employed this procedure to study the effects of repeated exposure to the popular recreational drug of misuse, (±)-3,4-methylenedioxymethamphetamine (MDMA) on the neuro-metabolome of rats. Repeated intermittent exposure to MDMA increased MDMA-produced locomotor hyperactivity and facilitated the acquisition of MDMA self-administration, indicating that repeated exposure had sensitized these behavioral responses (van de Wetering & Schenk, 2017). This was not due to changes in MDMA-induced extracellular concentrations of serotonin (5-HT) or MDMA in the striatum, suggesting that other neurochemical mechanisms may be more important for the development of sensitization to the effects of MDMA (van de Wetering et al., 2022). In the current study, we use this untargeted, behavioral metabolomics procedure to identify other potential neurochemical correlates of sensitized MDMA-produced behavior following repeated exposure in rats.

## Methods

In this study, untargeted metabolomics analysis was carried out on liquid chromatography–mass spectrometry (LC-MS) data obtained from 336 microdialysis samples collected during a previously conducted behavioral sensitization experiment in rats (van de Wetering et al., 2022). The animal treatment and sample collection/analysis methods are summarized below.

### Animals, treatments, and microdialysis

As previously reported (van de Wetering et al., 2022), adult, male Sprague-Dawley rats (*n* = 21) were stereotaxically implanted with intracerebral guide cannula (9.14. IC, Microbiotech, Sweden) in the medial striatum. One week later, rats began an 8-day MDMA sensitization experiment. On days 1–5, rats were placed into locomotor activity chambers (42 × 42 × 30 cm; Med Associates Inc., USA; model ENV-515) for 30 min prior to receiving an intraperitoneal (i.p.) injection of physiological saline (*n* = 11) or 10 mg/kg MDMA (*n* = 10) and remained in these chambers for an additional 60 min thereafter. This pre-treatment regimen was used as it has previously been shown to reliably induce behavioral sensitization as well as enhance the acquisition of MDMA self-administration (van de Wetering & Schenk, 2017; Wetering & Schenk, 2020). On the 8th day, microdialysis probes (MAB 9.14.3, Microbiotech, Sweden) were inserted into the guide cannula and rats were returned to the locomotor activity chambers. After a 3-hour stabilization period, microdialysis samples were collected at 30 min intervals for 8 h using a microinfusion pump (HD 2000 infusion, Harvard Apparatus) with a flow rate of 0.5 µL/min. During this time, all subjects received ascending doses of MDMA (0.0, 5.0, 10.0, mg/kg, i.p.) at 2-hour intervals. Locomotor activity was simultaneously recorded throughout and summed into 30 min intervals. These doses of MDMA produce moderate levels of locomotor activity following acute exposure and behavioral sensitization in response to these doses was apparent in MDMA pre-treated animals (van de Wetering & Schenk, 2017; Wetering & Schenk, 2020).

### Sample preparation and LCMS analysis

As previously reported (van de Wetering et al., 2022), 2.5 µL of internal standard (100 nM D_2_-5-HT), 7.5 µL of borate buffer (sodium tetraborate, 100 nM), and 7.5 µL of BzCl (diluted to 5% in acetonitrile) was added to each 15 µL microdialysate sample, with mixing in between each addition, and then stored at -80 °C until analysis. Analysis was carried out using 30 µL of derivatized sample injected onto a Poroshell 120 SB-Aq 2.7 μm column (2.1 × 100 mm; Agilent Technologies) installed in an Agilent Technologies (Santa Clara, CA) 1260 HPLC connected to a 6530 quadrupole time-of-flight (Q-TOF) LC-MS equipped with a Jet-Stream electrospray ionization (ESI) source, using settings as previously described (van de Wetering et al., 2022).

### Data processing

LC-MS metabolomics data were processed using the open-source software, MZmine (v2.51) (Li et al., 2018; Myers et al., 2017; Pluskal et al., 2010). Data processing parameters are shown in Table 1. Parameters were optimized to ensure accurate automated separation and integration of the internal standard (D_2_-5-HT) as well as some known analytes (5-HT, MDMA) that were quantified using targeted methods in our previous study (van de Wetering et al., 2022). Features that were not detected in at least 50% of samples were excluded from further analysis. The final feature list contained 737 features.

### Statistical analysis

Principal components analysis (PCA) was used to provide an initial overview of the dataset. Because PCA is an unsupervised method that aims to explain the maximum variance in the data, differences between two treatments groups (e.g., drug-treated vs. drug naïve) will only be revealed if the between-group variance exceeds the within-group variance, which is often not the case with untargeted metabolomics data (Álvarez-Sánchez et al., 2010; Álvarez-Sánchez et al., 2010; Worley and Powers, 2013). Furthermore, our data set contains group × time-course metabolomics and behavioral data, which no single analysis is capable of fully describing.

Therefore, a two-step analytical procedure was carried out to determine which metabolites are of interest. First, in order to identify metabolites that were associated with changes in behavior produced by acute drug exposure, an orthogonal partial least squares (OPLS) model was fitted with the *Y* variable coded as the measured locomotor response and the relative concentrations of each detected feature/metabolite as *X* variables (Trygg & Wold, 2002; Wold et al., 1998). Variable importance on projection (VIP) scores were then used to determine which variables have the greatest impact on the model, with 1 being the threshold value (Galindo-Prieto et al., 2014, 2015). Second, in order to determine which metabolites were impacted by repeated drug exposure and differed as a function of MDMA pre-treatment group, 2 (pre-treatment group: saline, MDMA) × 16 (time: 0–480 min) analysis of variance (ANOVA), with time as a repeated measure, were carried out on select metabolites of interest (as determined by step one), followed by Šidák-corrected multiple comparisons.

All metabolomics data were normalized to the internal standard peak area. For multivariate analyses, data were log-transformed in order to achieve a normal distribution, and Pareto scaled so as to avoid highly abundant exogenous compounds such as MDMA from dominating the statistical models, as is recommended for MS metabolomics data (Livera et al., 2015; Sysi-Aho et al., 2007; van den Berg et al., 2006; Veselkov et al., 2011; Wheelock & Wheelock, 2013). Multivariate analyses were carried out using Soft Independent Modelling of Class Analogies (SIMCA; v17, Umetrics, Umeå Sweden). Both PCA and OPLS models were autofitted by SIMCA, and the default K-fold cross-validation procedure was performed. The overall fit of the model was determined by examining the cumulative R^2^*X* and R^2^*Y*, which represents the fraction of explained variation in *X* and *Y*, respectively. The predictive power of the model was assessed by examining Q^2^, which represents the fraction of variation explained by the cross-validated model. R^2^ and Q^2^ values that are relatively similar and above 0.5 represent a good model (Szymańska et al., 2012; Triba et al., 2015; Wheelock & Wheelock, 2013). Univariate testing was carried out using GraphPad Prism (v9.1.0, GraphPad Software, San Diego, California USA), with the level of significance set as *p* < .05.

### Metabolite annotation and LC-MS/MS

Select metabolites of interest were identified or annotated using external standards or targeted LC-MS/MS, respectively. For LC-MS/MS, microdialysate samples were pooled and 10 µL was analyzed in duplicate using the same chromatography method as previously described (van de Wetering et al., 2022) and the following targeted Q-TOF ESI-MS/MS parameters: positive ion mode; gas temperature, 275 °C; gas flow, 8 L/m; nebulizer, 30 psi; capillary voltage, 2750 V; nozzle voltage, 0 V; fragmentor voltage, 130 V; acquisition rate, 2 spectra/s; mass range, m/z 50–500; collision-induced dissociation energies, 10, 20, and 40 eV. The resulting fragmentation patterns were compared to those recorded in the METLIN database (Smith et al., 2005) and with two previous studies that have characterized the MS/MS fragmentation of several BzCl-derivatized metabolites in microdialysis samples (Song et al., 2012; Wong et al., 2016).

**Table 1:** MZmine data processing parameters

Mass detection (centroid):	
	Noise level: 10^4^
Chromatogram builder (ADAP):	
	Minimum group size in # scans: 3
	Group intensity threshold: 10^4^
	Minimum highest intensity: 10^4^
	m/z tolerance: 0.02 m/z
Deconvolution (local minimum search):	
	m/z center calculator: median
	Chromatographic threshold: 35%
	Search minimum in RT range: 0.01 min
	Minimum relative height: 5%
	Minimum absolute height: 10^4^
	Minimum ratio of peak top/edge: 2
	Peak duration range: 0.02–0.5 min
Isotope peak grouper:	
	m/z tolerance: 0.005 m/z
	RT tolerance: 0.01 min absolute
	Monotonic shape: yes
	Maximum charge: 2
	Representative isotope: lowest m/z
Alignment (join aligner):	
	m/z tolerance: 0.02 m/z
	Weight for m/z: 3
	RT tolerance: 0.25 min absolute
	Weight for RT: 2
Gap-filling (same RT and m/z range):	
	m/z tolerance: 0.01 m/z

## Results

**Fig. 1:**
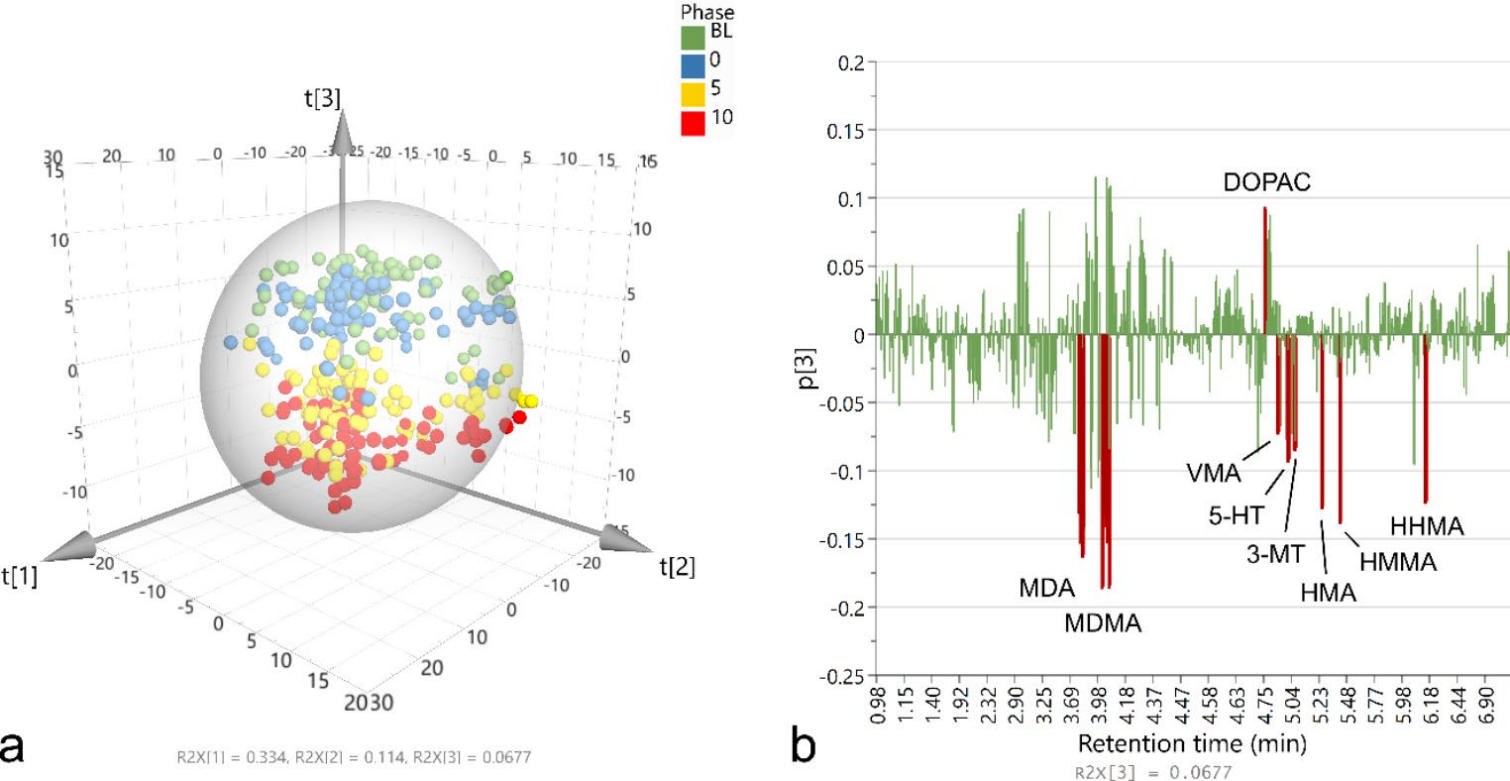
Results of PCA on LC-MS metabolomics data collected from 336 microdialysis samples. **a**) 3D scores plot of the first three components with samples shaded according to acute MDMA dose (baseline [BL; green], 0.0 [blue], 5.0 [yellow], 10.0 [red] mg/kg). Ellipse/sphere represents Hotelling T2 (95%). **b**) Histogram showing variable loadings for component 3 as a function of retention time. R^2^X values of each component are shown at the bottom of each graph

An overview of the full data set (737 features detected across 336 samples) was provided by PCA (R^2^*X* = 0.91, Q^2^ = 0.85). Figure 1 A shows the resulting 3D scores plot, which plots each sample’s score on the first 3 components (T[1–3]). Here, samples that cluster together represent those with a similar metabolomic profile. Some minor outliers were present, as determined by the ellipse/sphere representing the 95% confidence interval for Hotelling’s *T*2 test. No grouping was apparent on components 1 or 2 based on any experimental manipulation indicating that the primary variation in the dataset was uninduced. Clear grouping of the data along component 3 was observed based on acute MDMA dose, however. Figure 1B shows a histogram plotting the loading of each variable on component 3 (p[3]) as a function of LC-MS retention time. Several variables had a strong loading on component 3 and were driving the aforementioned grouping of samples based on acute MDMA dose. These included MDMA itself, 3,4-methylenedioxyamphetamine (MDA), 3,4-dihydroxymethamphetamine (HHMA), 4-hydroxy-3-methoxymethamphetamine (HMMA), 4-hydroxy-3-methoxyamphetamine (HMA), 5-HT, 3-methoxytyramine (3-MT), vanillylmandelic acid (VMA), and 3,4-dihydroxyphenylacetic acid (DOPAC). Note that the highly abundant exogenous compounds such as MDMA and MDA had multiple detected adducts and dimers (e.g., M + Na, 2M + H, 2M + Na etc.) as well as several fragments generated from in-source fragmentation (e.g., *m/z* 105 – benzoyl fragment).

**Fig. 2:**
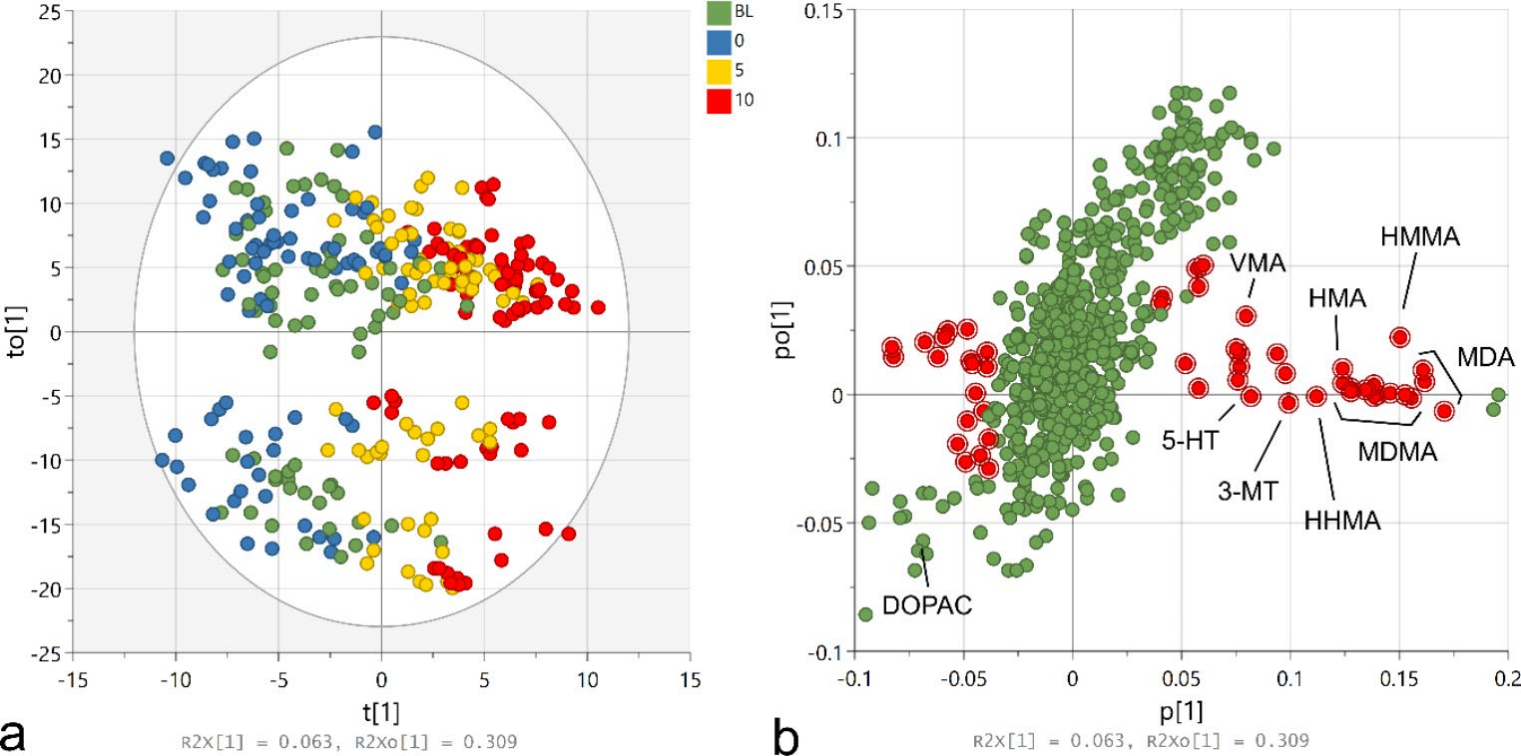
Results of the OPLS model assessing variables predictive of locomotor activity. **a**) Scores plot showing samples shaded according to acute MDMA dose (baseline [BL; green], 0.0 [blue], 5.0 [yellow], 10.0 [red]). Ellipse represents Hotelling T2 (95%). **b**) Loadings plot with variables of interest highlighted in red as determined by predictive-VIP scores > 1 and orthogonal-VIP scores < 1. R^2^X values of each component are shown at the bottom of each graph

**Fig. 3:**
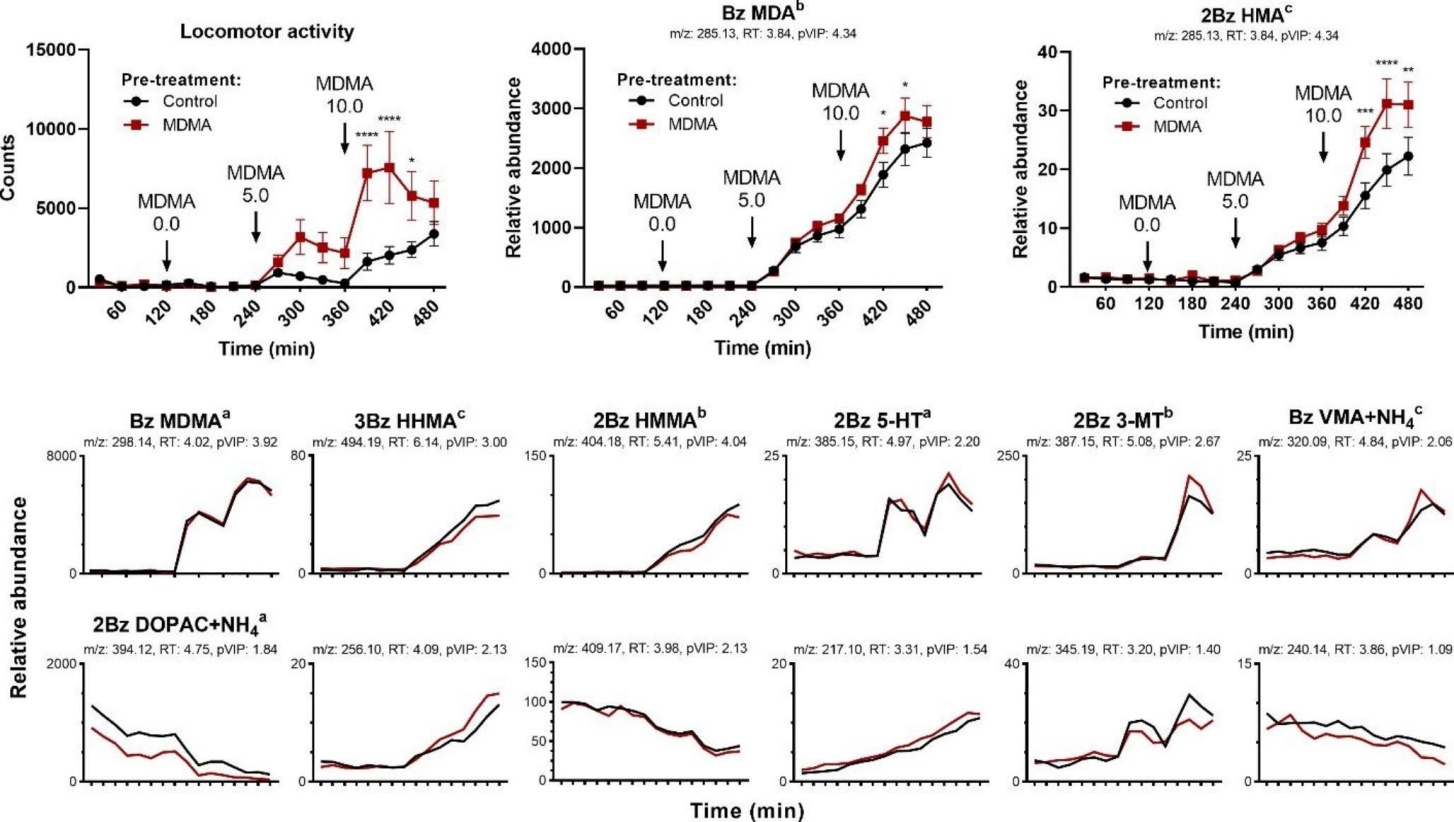
Mean (± standard error of the mean) locomotor activity and relative concentration of select metabolites of interest as a function of time and MDMA pre-treatment group. MDMA 0.0, 5.0, and 10.0 mg/kg i.p. was administered at 120, 240, and 360 min, respectively. ^a^ID based on external standards. ^b^ID based on MS/MS fragmentation data. ^c^ID based on mass and adduct pattern. *p < .05, **p < .01, ***p < .001, ****p < .0001 compared to control; two-way ANOVA followed by Šidák-corrected multiple comparisons

In order to determine which metabolites were dose-dependently associated with acute, MDMA-produced behavior over time, an OPLS model was fitted to the full data set with the *Y* variable coded as the locomotor activity (R^2^*Y* = 0.60, R^2^*X* = 0.57, Q^2^ = 0.46). Figure 2 A shows the resulting scores plot, where t[1] represents the predictive component and to[1] represents the first orthogonal component. Clear grouping of samples according to acute MDMA dose was observed along the predictive component as well as large orthogonal variation. Figure 2B shows the loadings plot, where each variable is plotted as a function of its predictive (p[1]) and orthogonal (po[1]) loadings. The variables that were the most strongly predictive of locomotor activity while also having little orthogonal variation, as determined by having predictive VIP scores above 1 and orthogonal VIP scores below 1, are highlighted in red. Figure 3 shows a summary of the metabolites of interest as identified by the OPLS model using these criteria, after filtering for non-unique metabolites (i.e., adducts, fragments) and a cluster of metabolites whose relative concentrations were impacted by ion suppression due to co-elution with the highly abundant MDMA. Of the metabolites/compounds that were detected in our samples, the strongest predictors of MDMA-induced locomotor activity were MDA, followed by MDMA, HMMA, 3-MT, 5-HT, and VMA. DOPAC was also a strong negative predictor of locomotor activity (VIP = 1.84) but had high orthogonal variation. Several unidentified/annotated metabolites were also predictive of locomotor activity.

To determine which of these metabolites were impacted by repeated MDMA exposure, separate two-way ANOVAs (treatment × time) followed by Šidák-corrected multiple comparisons were used to assess changes in the relative concentration of these metabolites of interest as a function of MDMA pre-treatment group. Significant interaction effects were found for MDA, *F*(15, 285) = 1.87, *p* = .0256, and HMA, *F*(15, 285) = 4.08, *p* < .0001, and multiple comparisons indicated significantly higher MDMA-produced MDA and HMA concentrations were present in the MDMA pre-treated group at later time points (*p* < .05; Fig. 3).

### Metabolite annotation

**Fig. 4:**
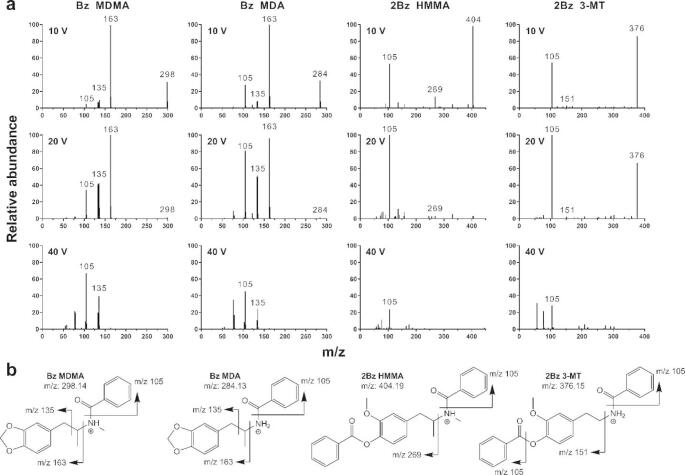
**a**) Q-TOF-ESI MS/MS spectra of benzoylated MDMA, MDA, HMMA, and 3-MT at 10, 20, and 40 eV collision energies. **b**) Proposed fragmentation of MDMA, MDA, HMMA, and 3-MT

Ions detected at *m/z* 298.14, 385.15, and 394.12 were identified as the protonated molecular ions of benzoylated MDMA, 5-HT, and DOPAC + NH_4_, respectively, with the use of external standards. Targeted MS/MS was used to annotate other metabolites of interest in pooled microdialysate samples (Fig. 4). The ion at *m/z* 384.13 produced a fragmentation pattern similar to MDMA and was annotated as MDA, while *m/z* 404.19 was annotated as HMMA, having produced a unique fragment at *m/z* 269.12, which would be expected if it fragmented in the same location as both MDMA and MDA. The ion detected at *m/z* 376.15 was annotated as benzoylated 3-MT; the primary product was the benzoyl fragment (*m/z* 105) while the most abundant unique fragment was at *m/z* 151.07, which is consistent with previous reports (Wong et al., 2016) and the METLIN database. No conclusive MS/MS fragmentation data were obtained for other metabolites of interest, but *m/z* 494.19 and 390.17 were suspected to be benzoylated HHMA and HMA, respectively, based on their mass and adduct pattern. Lastly, *m/z* 320.09 was suspected to be benzoylated VMA + NH_4_ based on previous reports (Wong et al., 2016).

## Discussion

In the current study, we designed a new method to improve the translatability of preclinical metabolomics research and help filter through potentially overwhelming and often noisy datasets. Using a combination of microdialysis and behavioral measures, we show a small number of behaviorally relevant changes in metabolites that are impacted by MDMA exposure.

**Fig. 5:**
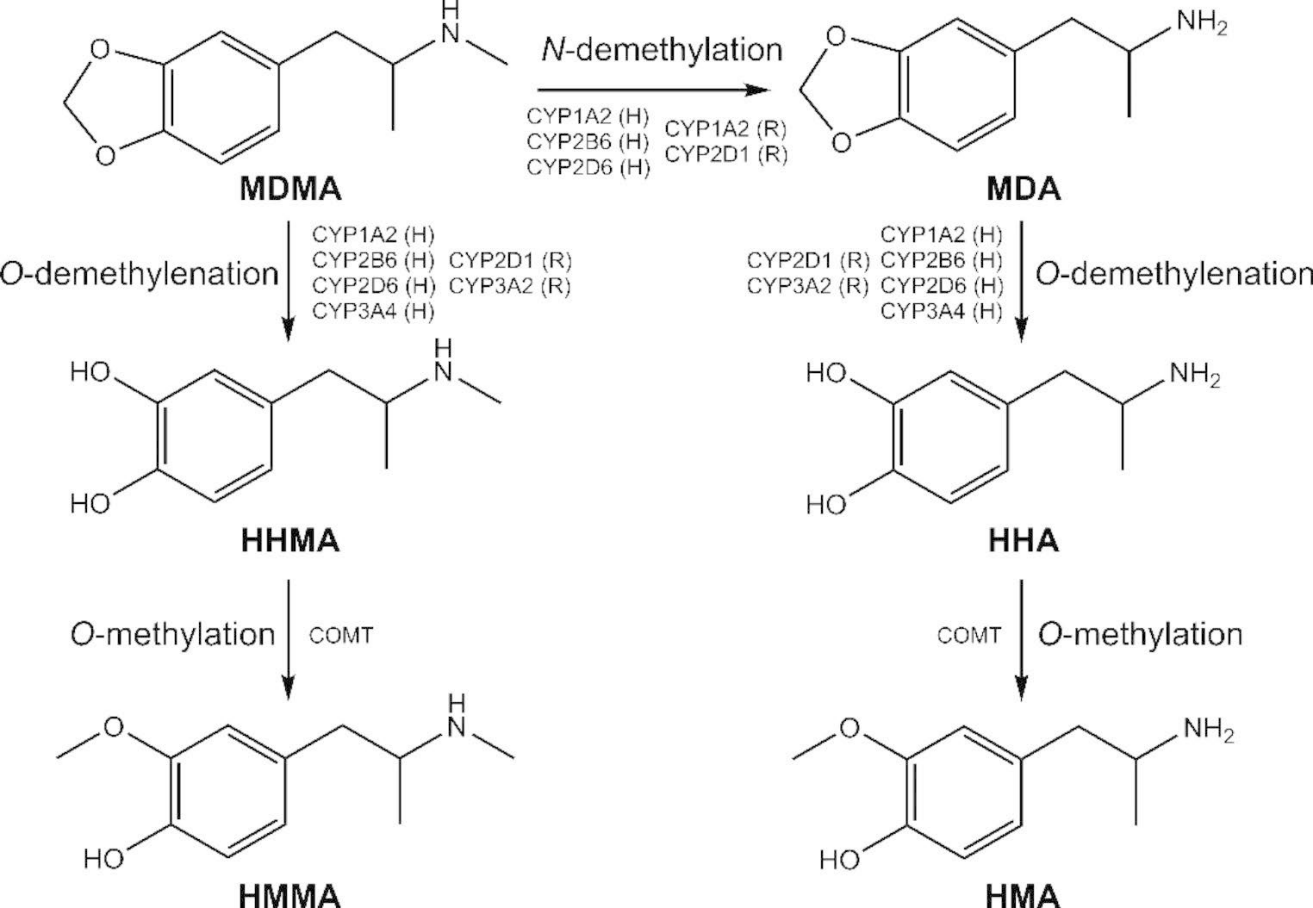
Primary metabolic pathways of MDMA and the CYP enzymes involved in both rats (R) and humans (H) (de la Torre & Farré, 2004)

In the first step of our analysis, we fitted an OPLS model with locomotor activity as the *Y* variable and the metabolomics data as *X* variables. By doing so, we were effectively able to determine which metabolites were most associated with dose-dependent increases in MDMA-produced locomotor activity over time. MDMA and its derivatives, MDA, HMA, HHMA, and HMMA (Fig. 5), were among the highest predictors of MDMA-induced locomotor activity, as was expected. MDMA is a potent psychostimulant (Cole & Sumnall, 2003). It induces the release and prevents the reuptake of the monoamine neurotransmitters, 5-HT, dopamine (DA), and norepinephrine (NE) (Green et al., 2003), all of which have well-established locomotor-activating effects (Ball et al., 2003; Baumann et al., 2008b; Berger et al., 1992; Bubar et al., 2004; Callaway et al., 1990, 1992; Daniela et al., 2004; Fantegrossi et al., 2004; Gold et al., 1989; Hekmatpanah & Peroutka, 1990; Kehne et al., 1996; Selken & Nichols, 2007). In line with this, our results indicated that 5-HT and several DA/NE-derived metabolites including 3-MT, VMA, and DOPAC, were also important predictors of MDMA-induced locomotor activity, though we were not able to directly detect DA or NE in our samples due to methodological parameters. The relative concentrations of these metabolites were all associated with dose-dependent increases in MDMA-induced locomotor activity, either positively or negatively, and changed as a function of acute MDMA administration in a manner consistent with previous quantitative studies (Baumann, Clark, & Rothman, 2008; Baumann, Clark, Franken, Baumann et al., 2008a, b; Bradbury et al., 2013; Colussi-Mas et al., 2010; Fernández-Galaz et al., 1993; Gough et al., 2006; Kankaanpää et al., 1998; Kurling et al., 2008; Morales-Villagrán et al., 1999; Nash, 1990; O’Shea et al., 2005).

Of the 700 + features detected in our samples, approximately 50 were associated with MDMA-produced behavior, and fewer than 20 of these were unique metabolites/compounds. Given that our data set was already reduced to a manageable size, we proceeded to use univariate statistics in our second analytical step, which aimed to evaluate these ~ 20 behaviorally relevant metabolites for differences as a function of MDMA pre-treatment group and identify those that were also impacted by repeated MDMA exposure^1^. Our results suggest potential changes in MDMA metabolism. Although the concentration of MDMA itself did not change as a function of repeated MDMA exposure, the concentration of both MDA (a primary MDMA metabolite) and HMA (a metabolite of MDA) were significantly higher in MDMA pre-treated rats compared to drug naïve controls. These results were mirrored, albeit non-significantly, by decreases in HHMA (a primary MDMA metabolite) and HMMA (a metabolite of HHMA). Together, these results might suggest an increase in MDMA *N*-demethylation relative to other metabolism pathways as a function of repeated MDMA exposure (Fig. 5) and could be due to changes in cytochrome P450 (CYP) enzymatic activity associated with each metabolic pathway (de la Torre et al., 2004; de la Torre & Farré, 2004).

While most metabolites of MDMA (including HMA) have been shown to have little direct effect on locomotor activity (Schindler et al., 2014; Yeh & Hsu, 1991), MDA is a particularly potent psychostimulant and has been shown to be even more effective at stimulating locomotor activity than equivalent doses of MDMA (20 mg/kg i.p.; Bexis and Docherty, 2006). MDA also has greater potency to increase synaptic overflow of DA compared to MDMA (Johnson et al., 1986; Nash & Nichols, 1991). DA is an important driver of locomotor activity, and it is well-established that the development of sensitized behavioral responses to psychostimulants, including MDMA, are driven by sensitized DAergic mechanisms (Ball et al., 2006, 2009; Bradbury et al., 2012; Kalivas et al., 1998; Morgan et al., 1997; van de Wetering & Schenk, 2017; Vanderschuren & Kalivas, 2000; Vezina, 2007). Although we were not able to detect DA itself in our samples, we did see trending effects of repeated MDMA exposure on both 3-MT and DOPAC (both primary DA metabolites), as well as VMA (an end-stage catecholamine metabolite) that would be consistent with a sensitized DAergic response. Our findings, therefore, suggest that behavioral sensitization to MDMA may, in part, be driven by increased MDA concentrations as a function of repeated MDMA exposure and the more potent effect of MDA on DAergic mechanisms associated with psychostimulant sensitization.

We have previously shown that rats that develop sensitization to the locomotor activating effects of MDMA, also develop sensitization to the rewarding effects of MDMA, as indicated by significant increases in both the rate and proportion of rats that acquire MDMA self-administration (van de Wetering & Schenk, 2017). Sensitization to the effects of psychostimulants drugs has long been suggested as an important factor underlying SUDs (Robinson & Berridge, 1993; Vanderschuren & Pierce, 2010; Vezina, 2007). Thus, the current findings provide valuable insight on the mechanisms underlying the development of sensitization to the rewarding effects of MDMA and suggest that increased turnover of MDMA into MDA as a function of repeated exposure might increase the abuse liability of the drug. These findings contribute to the growing area of research that focusses on the development of pharmacokinetics-based treatments for SUDs (Gorelick, 2012; Lin et al., 2021; Zheng et al., 2022).

It should be noted that the metabolism of MDMA is complex, however. Although MDMA metabolism is qualitatively similar across different animal species, and involves the same primary metabolic pathways and homologous CYP isoenzymes (Fig. 5), these enzymes are not functionally identical, and can have different Vmax values resulting in different metabolic pathways being prevalent at different doses in different species (Bogaards et al., 2008; de la Torre & Farré, 2004). MDMA can also inhibit its own metabolism, through the formation of complexes with CYP enzymes (de la Torre et al., 2000; Delaforge et al., 1999). This may further interact with genetic polymorphisms in CYP enzymes, which have been shown to impact the metabolism of MDMA into MDA in humans (de la Torre et al., 2012; Vizeli et al., 2017). Finally, to add to this complexity, our findings suggest that the metabolism of MDMA can change as a function of repeated exposure. Therefore, future studies using targeted, hypothesis-driven approaches are needed to clarify the role of MDMA metabolism, MDA, and DA in the development of sensitization to the effects of MDMA and the impact of this on MDMA use disorders in humans.

### Limitations

There are some limitations to the current study that should be discussed. Firstly, the derivatization and LC-MS parameters were optimized for the targeted quantification of 5-HT and MDMA for a previous, hypothesis-driven study (van de Wetering et al., 2022), and not for untargeted metabolomics. Metabolomics analysis of the samples was retrospective and as a result, the dataset was noisy and limited; several relevant metabolites (e.g., DA) that should be detectable in BzCl derivatized microdialysate samples were not detected (Song et al., 2012; Wong et al., 2016), and there was a large amount of uninduced variation. Nevertheless, the study served as a proof of concept. The use of supervised OPLS helped to identify behaviorally relevant results amongst significant noise. With some optimization of the derivatization and LC-MS procedures for untargeted metabolomics, detection of a wider range of metabolites could be achieved in order to cast a larger ‘metabolomic net’ and generate a larger dataset for analysis. Secondly, while microdialysis offers several advantages as a neurochemical sampling procedure, it is technically challenging due to the low sample volumes obtained and low concentrations of metabolites (for reviews see van Mever et al., 2021; Zestos & Kennedy, 2017). To overcome this, we adapted BzCl derivatization procedures from a previous targeted study in order to increase metabolite stability and improve detection limits (Song et al., 2012; Wong et al., 2016). Of course, the issue with derivatization and untargeted metabolomics is metabolite identification. BzCl reacts with both primary and secondary amines, phenols, and ribose-hydroxyl groups (Song et al., 2012; Wong et al., 2016). Because BzCl derivatization adds a varying number of benzoyl groups to metabolites, determination of the original mass becomes challenging, and the results cannot be simply compared to large databases. A potential means to address this issue is to use elementally labelled BzCl (i.e., bromo- or chloro-benzoyl chloride), which could isotopically elucidate the number of BzCl adducts of each metabolite in order to more readily calculate the original mass for comparison against metabolomics databases (Castro-Falcón et al., 2016; Schäfer et al., 2023).

## Conclusions

There is an increasing interest in monitoring dynamic changes in the metabolome over time (Rusilowicz et al., 2018; Smilde et al., 2010). Microdialysis provides the means to achieve this in vivo by sampling central metabolites with relatively high temporal resolution when compared to other metabolomics sampling techniques. While a handful of studies have developed *targeted* metabolomics procedures using microdialysis (Bongaerts et al., 2018; Song et al., 2012; Wong et al., 2016), the current study is the first *untargeted* metabolomics study. This is also the first metabolomics study to collect and analyze behavioral data alongside metabolomics data. A commonly reported limitation of -omics research, including metabolomics, is the poor translation of results to the disease or disorder phenotype (Humer et al., 2020; Sethi & Brietzke, 2016; Wang et al., 2016). By utilizing behavioral models of psychiatric disorders, and incorporating the behavioral data into the metabolomics analyses, we show that it is possible to determine which metabolites are most associated with the behavior of interest. This novel approach will be applicable to the study of drug effects and other research areas in all subjects regardless of species, age, or sex, and can improve the relevance and translatability metabolomics research.

Statements and declarations.

## Data Availability

The availability of all data presented in this study is from authors upon request.

## Footnotes

1 With larger data sets, it would be recommended to first conduct supervised, class-based multivariate analyses (i.e., PLS-DA/OPLS-DA) and filter the results for behaviourally relevant metabolites before proceeding to any univariate analyses.

## Abbreviations

**3-MT**: 3-methoxytyramine
**5-HT**: Serotonin
**ANOVA**: Analysis of variance
**CYP**: Cytochrome P450
**DA**: Dopamine and
**DOPAC**: 3,4-dihydroxyphenylacetic acid
**ESI**: Electrospray ionization
**HHMA**: 3,4-dihydroxymethamphetamine
**HMA**: 4-hydroxy-3-methoxyamphetamine
**HMMA**: 4-hydroxy-3-methoxymethamphetamine
**HPLC**: High performance liquid chromatography
**LC-MS**: Liquid chromatography–mass spectrometry
**MDA**: 3,4-methylenedioxyamphetamine
**MDMA**: (±)-3,4-methylenedioxymethamphetamine
**NE**: Norepinephrine
**OPLS**: Orthogonal partial least squares
**PCA**: Principal components analysis
**Q-TOF**: Quadrupole time-of-flight
**SUD**: Substance use disorder
**VIP**: Variable importance on projection
**VMA**: Vanillylmandelic acid
